# Molecular Mechanisms of Oxidative Stress Relief by CAPE in ARPE−19 Cells

**DOI:** 10.3390/ijms24043565

**Published:** 2023-02-10

**Authors:** Changjie Ren, Peiran Zhou, Mingliang Zhang, Zihao Yu, Xiaomin Zhang, Joyce Tombran-Tink, Colin J. Barnstable, Xiaorong Li

**Affiliations:** 1 Tianjin Key Laboratory of Retinal Functions and Diseases, Tianjin Branch of National Clinical Research Center for Ocular Disease, Eye Institute and School of Optometry, Tianjin Medical University Eye Hospital, Tianjin 300384, China; 2 Department of Neural and Behavioral Sciences, Penn State College of Medicine, Hershey, PA 0850, USA

**Keywords:** CAPE, oxidative stress, ARPE−19 cells, UCP2

## Abstract

Caffeic acid phenylethyl ester (CAPE) is an antioxidative agent originally derived from propolis. Oxidative stress is a significant pathogenic factor in most retinal diseases. Our previous study revealed that CAPE suppresses mitochondrial ROS production in ARPE−19 cells by regulating UCP2. The present study explores the ability of CAPE to provide longer-term protection to RPE cells and the underlying signal pathways involved. ARPE−19 cells were given CAPE pretreatment followed by t-BHP stimulation. We used in situ live cell staining with CellROX and MitoSOX to measure ROS accumulation; Annexin V-FITC/PI assay to evaluate cell apoptosis; ZO−1 immunostaining to observe tight junction integrity in the cells; RNA-seq to analyze changes in gene expression; q-PCR to validate the RNA-seq data; and Western Blot to examine MAPK signal pathway activation. CAPE significantly reduced both cellular and mitochondria ROS overproduction, restored the loss of ZO−1 expression, and inhibited apoptosis induced by t-BHP stimulation. We also demonstrated that CAPE reverses the overexpression of immediate early genes (IEGs) and activation of the p38-MAPK/CREB signal pathway. Either genetic or chemical deletion of UCP2 largely abolished the protective effects of CAPE. CAPE restrained ROS generation and preserved the tight junction structure of ARPE−19 cells against oxidative stress-induced apoptosis. These effects were mediated via UCP2 regulation of p38/MAPK-CREB-IEGs pathway.

## 1. Introduction

Oxidative stress is involved in the pathogenesis of diverse retinal diseases, such as age-related macular degeneration (AMD), diabetic retinopathy (DR), glaucoma, retinal vascular occlusion, and inherited retinal diseases [1]. Reactive oxygen species (ROS) derived from molecular oxygen are produced by redox reactions or electronic excitation, which include free radicals, hydrogen peroxide, and oxygen ions from the byproducts of oxygen metabolism [2]. The richness of mitochondria in the retina generates sufficient ATP via oxidative metabolism to maintain its phototransduction and neurotransmission functions [3]. An imbalance between the oxidation and antioxidation systems in the retina can enhance the accumulation of ROS, and excessive levels of ROS lead to oxidative impairment of multiple types of retinal cells including retinal pigment epithelium (RPE) cells [4].

The RPE is a polarized monolayer of cells located between the neural retina and Bruch’s membrane [5]. In addition to absorbing light, transferring heat, metabolizing Vitamin A, and phagocytosing photoreceptor outer segments, the RPE also has a major function in transporting nutrients from the choriocapillaris to photoreceptors, which is indispensable for maintaining visual function [6]. Under normal physiological conditions the retina demands a higher oxygen supply. Intensive oxygen metabolism, continual exposure to light, high concentrations of polyunsaturated fatty acids, and the presence of photosensitizers increase ROS production in the retina [7]. The pivotal anatomy location and physiological function render RPE cells particularly vulnerable to oxidative injury.

Mitochondria play a crucial role in energy production through oxidative phosphorylation. Extensive literature supports the idea that mitochondria are the main source of ROS generation [8,9,10]. The proton gradient across the inner mitochondrial membrane is a key driving force for mitochondrial ROS production, and this gradient can be modulated by members of the mitochondrial uncoupling protein (UCP) family. Among the UCPs, UCP2 is expressed in most tissues and appears to be under the tightest regulation, making it a prime candidate as a therapeutic target to alleviate numerous diseases involving ROS across many tissues, including the eye. Multiple lines of evidence suggest that UCP2 suppresses oxidative stress to protect retinal cells [11,12,13]. In RPE cells, various factors are able to reduce oxidative damage by enhancing the expression or activity of UCP2. More direct evidence for the role of UCP2 comes from experiments in which the expression of UCP2 was enhanced by the neuroprotective pigment epithelium-derived factor (PEDF) and allowed the RPE cells to better resist oxidative stress [14,15,16]. Increased expression of UCP2 improved the ability of aged RPE cells to resist oxidative damage, and UCP2 was predicted to be a new protective target for RPE cells in neurodegenerative retinal diseases [17].

Caffeic Acid Phenylethyl Ester (CAPE) was originally defined as a constituent of the propolis of honeybee hives with a broad spectrum of biological properties including anti-pathogenic, anti-carcinogenic, anti-inflammatory, and immunomodulation and wound-healing activities [18]. A growing corpus of evidence suggests CAPE has a beneficial effect in some experimental retinal disease models. CAPE protects retinal neurons and attenuates inflammatory responses in a rat model of optic nerve crush [19]. CAPE treatment also decreases the oxidative stress levels in the retinas of the streptozotocin-induced diabetic rat model [20]. In our previous work, we found that CAPE is a potent regulator of acute oxidative stress. It acts primarily through UCP2 to protect retinal ganglion cells in an ischemia/reperfusion mouse model and resists LPS-induced oxidative damage in the adult retinal pigment epithelium (ARPE−19) cell line [21]. However, the ability of CAPE to provide longer-term protective effects and its underlying mechanism of action, in addition to lowering mitochondrial membrane potential, has not been elucidated yet.

In the present study, we examined the protective role of CAPE in t-BHP-induced oxidative injury in ARPE−19 cells and found that CAPE effectively combats t-BHP stimulation-induced cellular ROS generation for at least 12 h. We further explored the mechanisms of CAPE’s activity by analyzing and verifying RNA-seq data and found that CAPE could reverse abnormal expression of IEGs to physiological levels. Moreover, we demonstrated this regulation by CAPE was partly via the UCP2-mediated down-streaming signaling pathway. Based on these data, we propose that CAPE is a promising drug candidate to defend RPE cells against oxidative stress-induced cell death and barrier dysfunction in retinal diseases.

## 2. Results

### 2.1. Natural Compounds with Antioxidant Properties Inhibited t-BHP Induced ROS Production in ARPE−19 Cells

**Figure 1:**
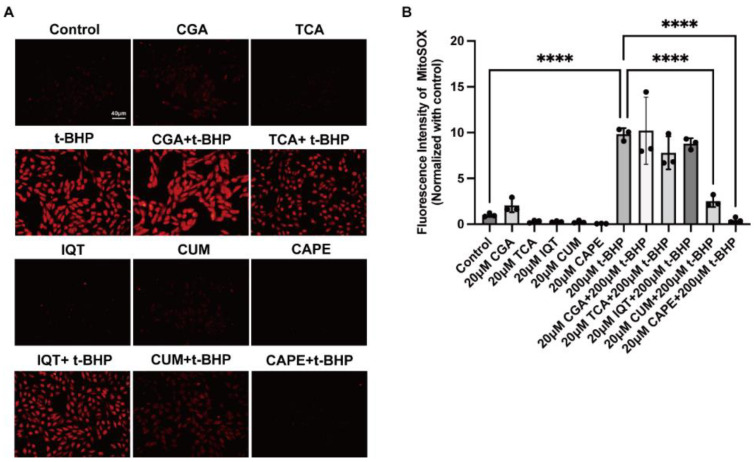
Treatment of ARPE−19 cells with CGA, TCA, IQT, CUM, or CAPE leads to inhibition of t-BHP-induced mitochondrial and cellular ROS generation. (**A**) Immunofluorescence microscopic images of live ARPE−19 cells stained with MitoSOX (red). Scale bar = 40 μm; (**B**) Quantification of MitoSOX immunofluorescence intensity; (**C**). Confocal images of live ARPE−19 cells stained with MitoSOX (red), CellROX (violet), and Hoechst (blue). Scale bar = 10 μm; Quantification of MitoSOX (**D**) or CellROX (**E**) immunofluorescence intensity. **** *p* < 0.0001. The data are presented as the mean ± SD, normalized to control, *n* = 3.

To determine whether the actions of CAPE in reducing oxidative stress were unique, we compared the effects of CAPE with a series of structurally related compounds. Chlorogenic Acid (CGA) [22], Trans-Cinnamic Acid (TCA) [23], Isoquercetin (IQT) [24], Curcumin (CUM) [25], and CAPE are natural compounds containing polyphenol structures, all of which have reported antioxidant properties. As shown in Figure 1A, we pretreated ARPE−19 cells with each of the compounds (20 μM) for 1 h, followed by stimulation with 200 μM t-BHP for 2 h, and then determined mitochondrial ROS levels using the MitoSOX assay. In Figure 1B, quantification of the fluorescence intensity of ROS (normalized to the control group) indicated that t-BHP dramatically increased mitochondrial ROS generation (9.82 ± 0.66-fold, *p* < 0.0001). Among the compounds tested, only CUM (2.51 ± 0.62-fold, *p* < 0.0001), and CAPE (0.47 ± 0.27-fold, *p* < 0.0001) inhibited mitochondrial ROS generation. while CGA, TCA, and IQT did not show significant inhibition.

We next tested whether CAPE blocked the production of both mitochondrial and cellular ROS. Treatment of ARPE−19 cells with 200 μM t-BHP at different time points (2 h, 6 h, 12 h) induced an increase in both mitochondrial (determined using MitoSox) and cellular (determined using CellROX) ROS production in a time-dependent manner (Figure 1C). By 12 h cells were dying, and nuclear staining was apparent. As shown in Figure 1D–E, both mitochondrial ROS (2.58 ± 0.23, *p* < 0.0001) and cellular ROS (9.72 ± 1.20-fold, *p* < 0.0001) were significantly increased after 12 h t-BHP stimulation compared to the control. CAPE significantly decreased both t-BHP-induced mitochondrial and cellular ROS production (1.23 ± 0.13, *p* < 0.0001 and 0.48 ± 0.01-fold, *p* < 0.0001, respectively) to near control levels.

### 2.2. CAPE Prevents t-BHP-Induced Apoptosis in ARPE−19 Cells

**Figure 2:**
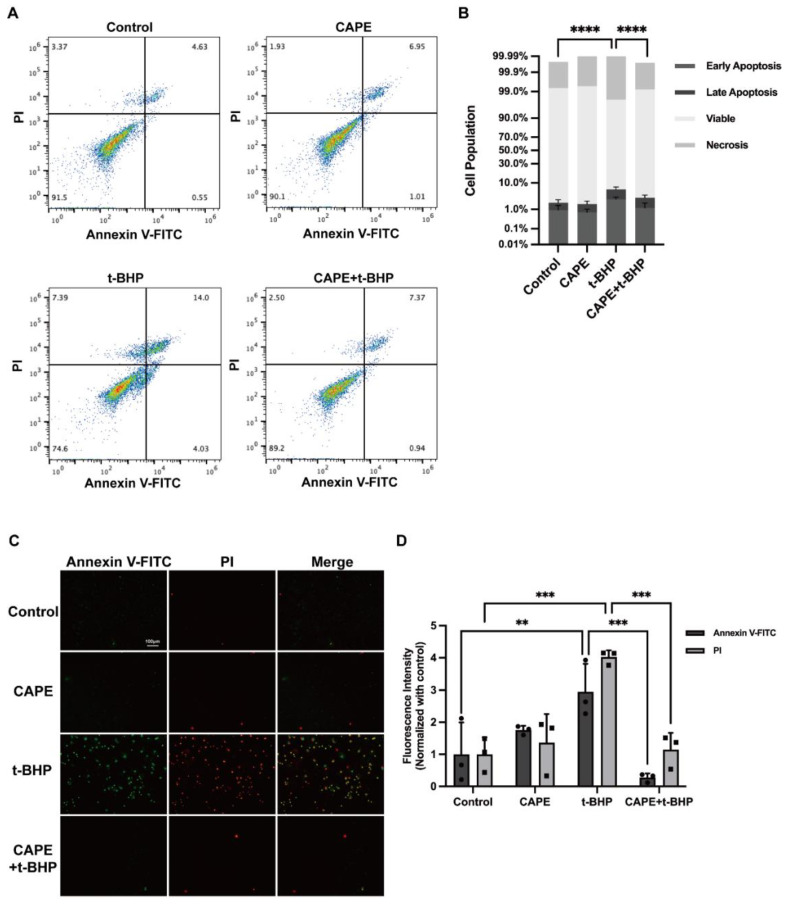
CAPE suppressed apoptosis in ARPE−19 cells exposed to t-BHP stimulation. (**A**) Flow cytometry after staining with both Annexin V-FITC and PI to assess; (**B**) Quantification of cell population in each treatment group. The proportion of non-apoptotic cells (lower left square: Annexin V-FITC^−^/PI^−^), early apoptotic cells (lower right square: Annexin V-FITC^+^/PI^−^), late apoptotic/necrotic cells (upper right square: Annexin V-FITC^+^/PI^+^) and dead cells (upper left square: Annexin V-FITC^−^/PI^+^); (**C**) Immunofluorescence microscopic images of ARPE−19 cells double-labeled with Annexin V-FITC (green) and PI (red): live cells (Annexin V-FITC^−^/PI^−^), early apoptotic cells (Annexin V-FITC^+^/PI^−^), late apoptotic cells (Annexin V-FITC^+^/PI^+^), dead cells (Annexin V-FITC^−^/PI^+^). Scale bar = 100 μm; (**D**) Image quantification of immunofluorescence intensity for Annexin V-FITC/PI. ** *p* < 0.01, *** *p* < 0.001, **** *p* < 0.0001. Values represent the mean ± SD, normalized with control, *n* = 3.

To test the effects of CAPE on apoptosis we pretreated ARPE-19cells with 20 μM CAPE for 1 h, followed by 200μM t-BHP stimulation for 12 h (Figure 2). Apoptosis was detected using an Annexin V-FITC/PI apoptosis detection kit and flow cytometry (Figure 2A,B). This assay measured both early apoptotic cells (lower right square) and late apoptotic cells (upper right square). The total percentage of apoptosis cells increased from 1.95 ± 0.22% (control) to 6.15 ± 1.03% after t-BHP stimulation, and this was significantly decreased by CAPE pretreatment (3.06 ± 0.65%). Correspondingly, the percentage of live cells (lower left square) was greater in the CAPE + t-BHP group (96.13 ± 0.61%) compared with the t-BHP group (91.60 ± 0.26%). In addition, apoptosis was monitored visually via Annexin V-FITC/PI staining (Figure 2C,D) to identify different cell populations. Positive staining of both early and late apoptotic cells was significantly increased after t-BHP stimulation, but CAPE pretreatment markedly reduced both groups of t-BHP induced apoptosis.

### 2.3. CAPE Inhibits t-BHP Induced Disruption of Tight Junction Protein ZO−1 in ARPE−19 Cells

**Figure 3:**
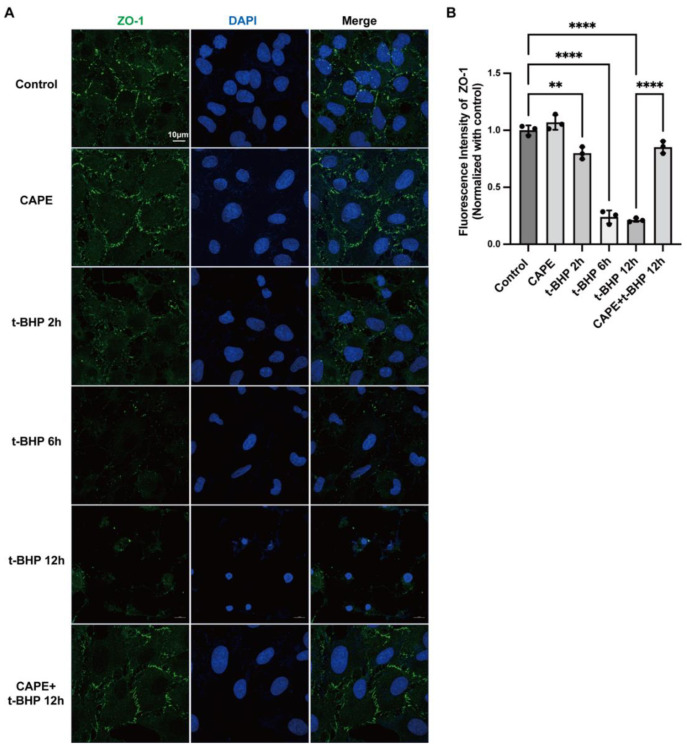
CAPE preserves expression of the ZO−1 tight junction protein in ARPE−19 cells exposed to t-BHP. (**A**) Confocal images of live ARPE−19 cells stained with ZO−1 (green) and DAPI (blue). Scale bar = 10μm; (**B**) Quantification of immunofluorescence intensity of ZO−1 expression in ARPE−19 cells. **p < 0.01, **** *p* < 0.0001. The data are presented as the mean ± SD, normalized to control, *n* = 3.

To determine whether CAPE treatment could maintain the barrier functions of RPE cells, we examined the tight junction protein ZO−1. ARPE−19 cells were stimulated by 200 μM t-BHP (2 h, 6 h, or 12 h) with or without CAPE pretreatment, and ZO−1 as an indicator of cell hyperpermeability was evaluated by immunofluorescence (Figure 3A,B). In contrast with the control, t-BHP significantly disturbed the integrity of ZO−1 in a time-dependent manner: the loss of ZO−1 could be observed at 2 h (0.80 ± 0.05-fold, *p* < 0.01), and worsened at 6 h (0.24 ± 0.06-fold, *p* < 0.0001), while at 12 h (0.21 ± 0.02-fold, *p* < 0.0001) almost no ZO−1 positive staining detected. Pretreatment by CAPE, however, preserved ZO−1 expression and morphology (0.85 ± 0.05-fold, *p* < 0.01) against t-BHP injury.

### 2.4. CAPE Modulates Oxidative Injury induced Gene Expression Profile Changes in ARPE−19 Cells

**Figure 4:**
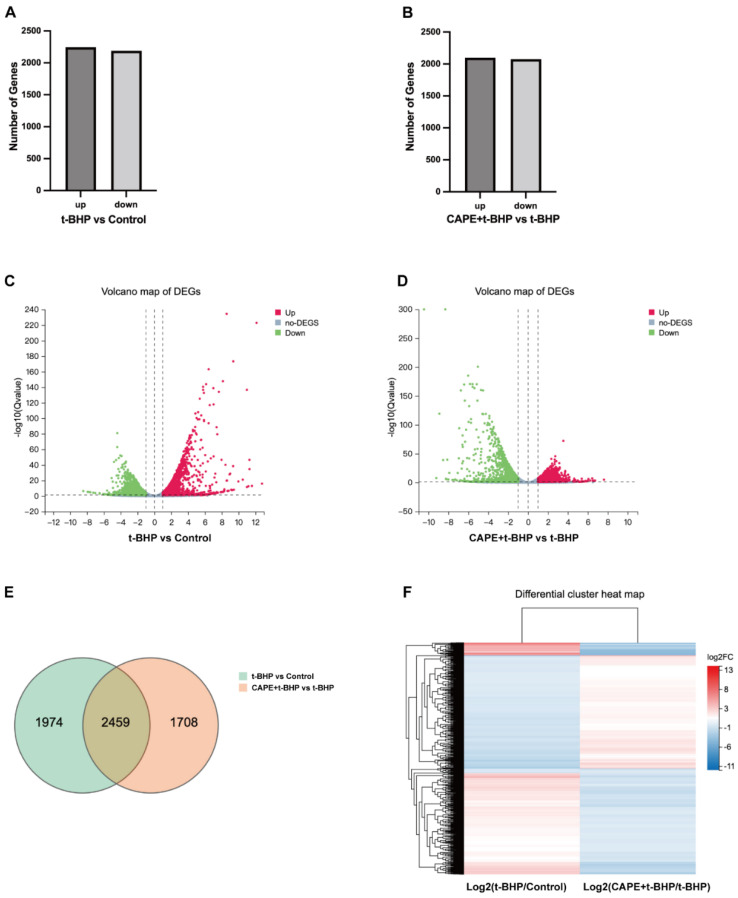

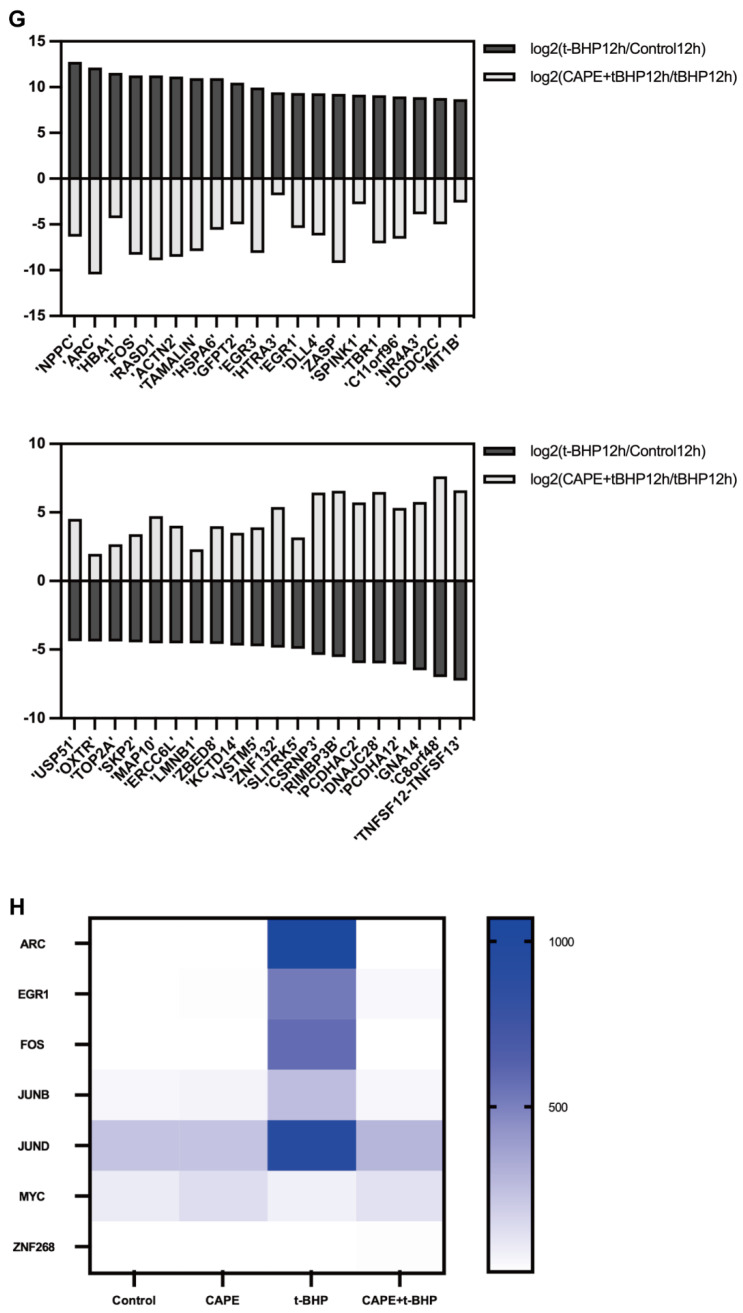
RNA-seq analysis of altered gene expression in CAPE pretreated ARPE−19 cells under t-BHP stimulation. (**A**,**B**) Overall changes in regulated DEGs after CAPE or t-BHP treatment (Q value < 0.05, |log2 Fold Change| > 1); (**C**,**D**) Volcano plots of DEGs after CAPE or t-BHP treatment: *X*-axis: fold change of the difference after conversion to log2, *Y*-axis: significance after conversion to log10, Red: DEG upregulated, Blue: DEG down-regulated, Gray: non-DEG (Q value < 0.05, |log2 Fold Change| > 1); (**E**) Venn of DEGs among groups. Each circle represents a group of gene sets. Areas superimposed by different circles represent the intersection of gene sets between the treatment groups. The non-overlapping sections of the circles indicate genes that are uniquely regulated in the indicated groups, (Q value < 0.05, |log2 Fold Change| > 1); (**F**) Heatmap of DEGs clusters between t-BHP/Control group and CAPE + t-BHP/t-BHP group (Q value < 0.05, |log2 Fold Change| > 1); (**G**) Top 20 most changed genes in both directions within 2459 overlapped DEGs among groups; (**H**) Heat map of IEGs expression in each group presented by the average transcripts per million (TPM) value.

After establishing that CAPE treatment attenuated t-BHP-induced oxidative injury in ARPE−19 cells, we next studied whether this treatment also showed more widespread effects. To examine this, we carried out RNA-seq on the CAPE pretreated oxidative stress in vitro model of ARPE−19 cells and 17,157 genes were detected in total. We found after t-BHP stimulation that the expression of 6316 genes was altered. With criteria Q value < 0.05, |log2 Fold Change| > 1, 2245 genes were upregulated and 2188 genes downregulated when compared to the control (Figure 4A,C). When the t-BHP group was compared to the CAPE + t-BHP group, there were 2097 genes upregulated and 2077 genes downregulated (Figure 4B,D). Among the differentially expressed genes (DEGs), 2459 showed overlapping expression changes in the t-BHP and CAPE + t-BHP groups. Expressions of 1974 genes were uniquely regulated by t-BHP and 1708 by CAPE + t-BHP compared to controls (Figure 4E). An unbiased cluster analysis of the overlapping 2459 DEGs suggested that multiple genes regulated by t-BHP were reversed in the direction of their expression by CAPE treatment (Figure 4F). The top 20 most extremely changed genes, and the effect of CAPE, are listed (Figure 4G). Although many groups of genes were affected by t-BHP and restored to near normal by CAPE, we focused on a group of immediate early genes (IEGs) as these could potentially have pleiotropic effects on later gene expression changes. Expression levels of ARC, EGR1, FOS, JUNB, JUND, MYC, and ZNF268 were identified in each of the treatment groups compared to controls (Figure 4H). ARC, EGR1, FOS, JUNB, and JUND were significantly upregulated under t-BHP stimulation and showed a negative relationship with CAPE treatment. Interestingly, other IEGs like MYC and ZNF268, t-BHP but not significantly influenced by CAPE pretreatment.

### 2.5. GO Enrichment Analyses of DEGs

**Figure 5:**
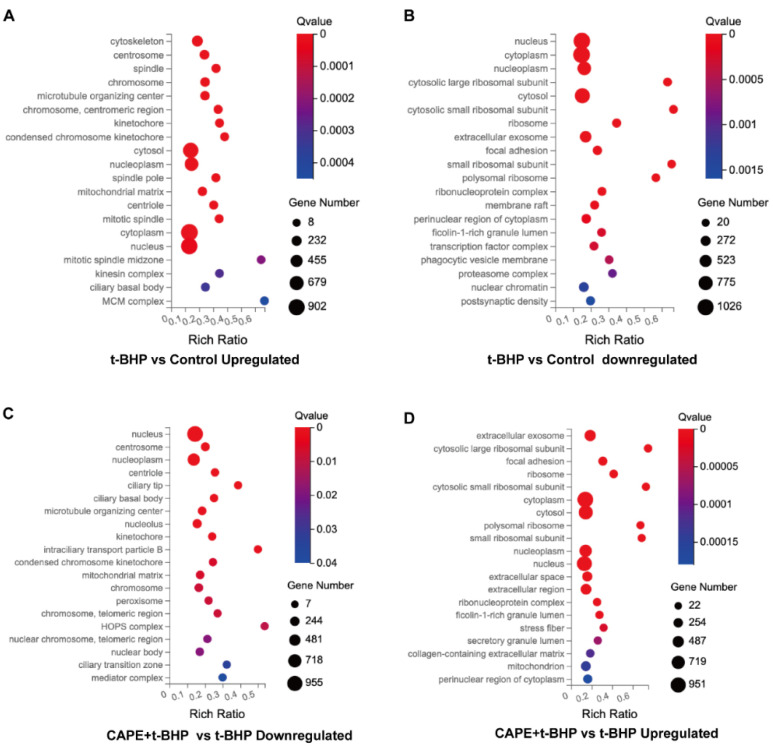
The top 20 GO enrichment of DEGs in cellular components. Upregulated (**A**) or Downregulated (**B**) t-BHP vs. control enrichment; Downregulated (**C**) or Upregulated (**D**) CAPE + t-BHP vs. t-BHP. *X*-axis: enrichment ratio (number of genes annotated to an entry in the selected gene set to the total number of genes annotated to the entry in the species, calculated as Rich Ratio = Term Candidate Gene Num/Term Gene Num). Size of circles: the number of DEGs annotated to a GO Term. Circle color: enriched significance. The redder the color, the smaller the significance value. Q-value ≤ 0.05 are considered as significant enrichment.

GO analysis was performed to cluster the DEGs (Figure S1), which were classified into three enriched categories: cellular component (Figure 5) molecular function, and biological process. In the cellular components’ enrichment, the top five highest categories in the identified upregulated DEGs in the t-BHP vs. control group were nucleus, centrosome, nucleoplasm, centriole, and mitochondrial matrix (Figure 5A). Correspondingly, DEGs, where CAPE pretreatment blocked upregulation by t-BHP, were also involved in the cellular components categories listed for the t-BHP vs. Control group (Figure 5C). Likewise, the downregulated DEGs in the t-BHP vs. control group enrichment (top five highest categories: nucleus, cytoplasm, nucleoplasm, cytosol, and extracellular exosome) correlated with the upregulated genes in the CAPE + t-BHP vs. t-BHP group (Figure 5B,D). Detailed results of the analysis are reported in Figures S2 and S3. Overall, these results show a widespread ability of CAPE to reverse the effects of the oxidative stressor t-BHP on gene expression.

### 2.6. qPCR Validation of Changes in the Immediate Early Genes (IEGs) in the RNA-seq Data

**Figure 6:**
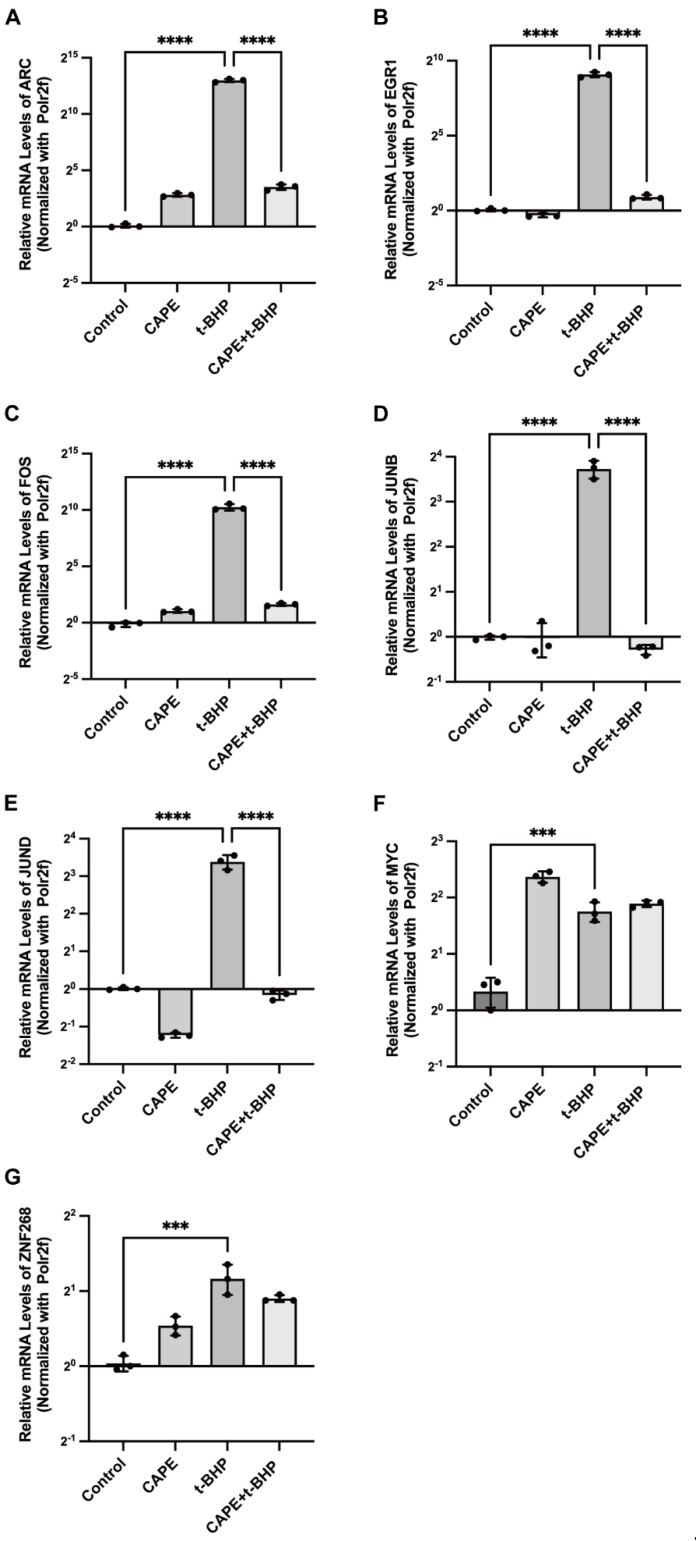
Pretreatment of ARPE−19 cells with CAPE before exposure to oxidative stress preserves normal expression of several IEGs. Relative mRNA expression levels of ARC (**A**), EGR1 (**B**), FOS (**C**), JUNB (**D**), JUND (**E**), MYC (**F**), ZNF268 (**G**) with each treatment. Experiments were repeated 3 times. IEGs expression was calculated by the 2^−△△Ct^ method and normalized to the internal reference Polr2f. *** *p* < 0.001, **** *p* < 0.0001.

Since IEGs can have substantial downstream effects, we investigated these further. We first confirmed the selected IEG results of the RNA-seq experiments using qPCR. The stimulation of ARPE−19 cells with t-BHP (200 μM) increases expression of almost all IEGs, including ARC (8141 ± 826.7-fold, *p* < 0.0001), EGR1 (539.8 ± 64.61-fold, *p* < 0.0001), FOS (1231 ± 241.5-fold, *p* < 0.0001), JUNB (13.24 ± 1.810-fold, *p* < 0.0001), and JUND (10.43 ± 1.391-fold, *p* < 0.0001). The expressions of these were all reversed by CAPE to near control levels (Figure 6A–E). It is worth noting that the levels of expression of several of these IEGs were reversed, by several to even thousands of folds. However, not all IEGs were regulated by CAPE. For example, the mRNA levels of MYC (3.364 ± 0.4077-fold, *p* < 0.001) and ZNF268 (2.240 ± 0.311-fold, *p* < 0.001) were upregulated after oxidative stress in the RPE cells, but CAPE did not reverse their expression levels (Figure 6F,G).

### 2.7. Effects of CAPE on t-BHP-Induced Expression of IEGs

**Figure 7:**
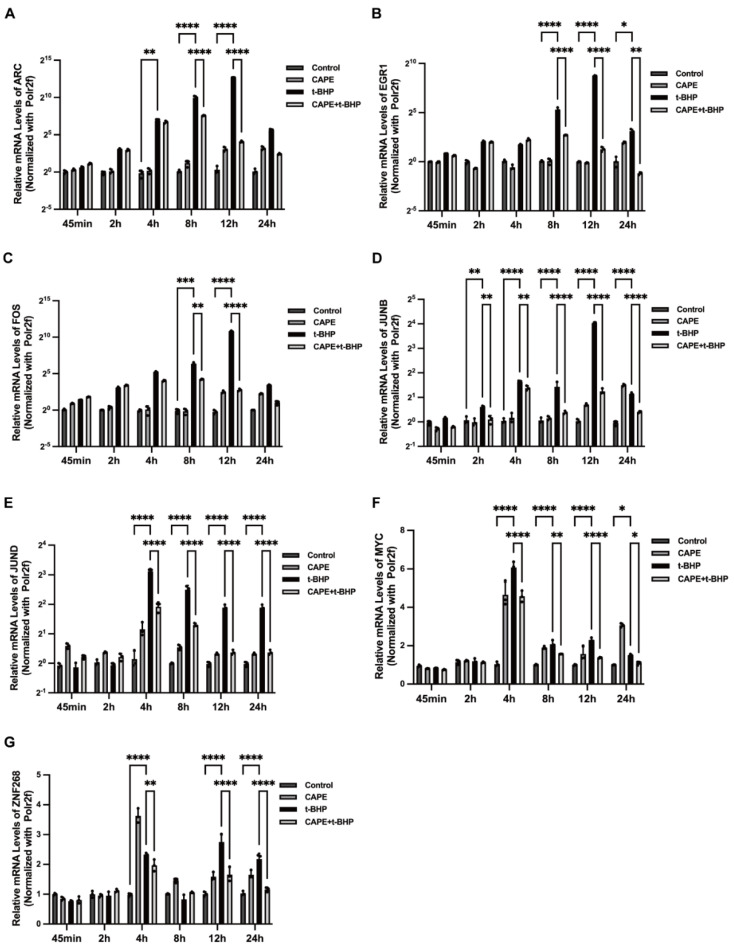
Changes in expression of IEGs correlate with CAPE pretreatment under varying t-BHP stimulation time. Relative mRNA expression levels of ARC (**A**), EGR1 (**B**), FOS (**C**), JUNB (**D**), JUND (**E**), MYC (**F**), ZNF268 (**G**) over 24 h. Experiments were repeated 3 times. IEGs expression was calculated by the 2^−△△Ct^ method and normalized to Polr2f. * *p* < 0.05, ** *p*< 0.01, *** *p* < 0.001, **** *p* < 0.0001.

We also analyzed the time course of IEGs’ expression after treatment with CAPE. Upregulation in expression of ARC, EGR1, FOS, or JUNB was detected at 4 h after exposure to t-BHP, peaking at 12 h, with expression differences among the IEGs (Figure 7). The effect of CAPE, however, was not immediate in reversing the t-BHP effect on these genes and only did so by 8 h and 12 h. Expression of JUND showed significant increases between 2 h−24 h in the oxidative stress environment with a peak at 4 h. Unlike the other IEGs, CAPE inhibited t-BHP-induced JUND overexpression as early as the 2 h time point.

### 2.8. UCP2 Mediates CAPE Regulation of IEGs

**Figure 8:**
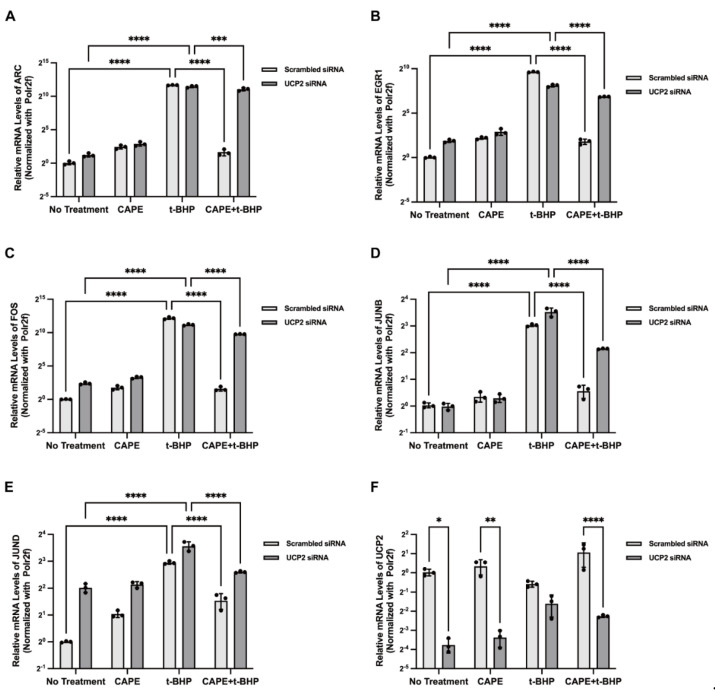
IEGs expression in ARPE−19 cells after UCP2 siRNA transfection. Relative mRNA expression levels of ARC (**A**), EGR1(**B**), FOS (**C**), JUNB (**D**), and JUND (**E**) in each group after 12 h t-BHP stimulation. The relative UCP2 mRNA expression levels among groups (**F**). Experiments were repeated 3 times. Relative mRNA expression was calculated using the 2^−△△Ct^ method and normalized to Polr2f. * *p* < 0.05, ** *p*< 0.01, *** *p* < 0.001, **** *p* < 0.0001.

**Figure 9:**
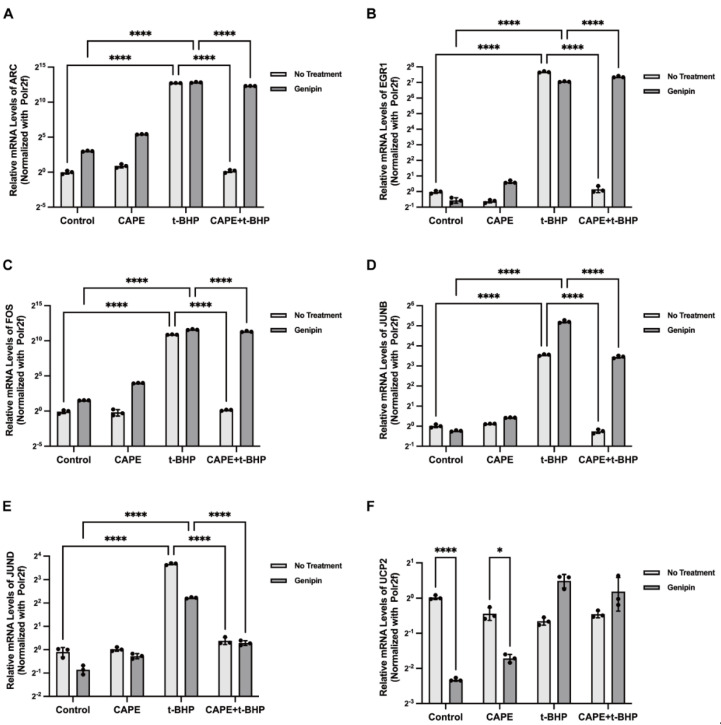
IEGs expression in ARPE−19 cells with Genipin treatment. The relative mRNA expression levels of ARC (**A**), EGR1(**B**), FOS (**C**), JUNB (**D**), and JUND (**E**) in each group after 12h t-BHP stimulation. The relative UCP2 mRNA expression levels among groups (**F**). Experiments were repeated 3 times. Relative mRNA expression was calculated by the 2^−△△Ct^ method and normalized to Polr2f. * *p* < 0.05, **** *p* < 0.0001.

In previous studies, we showed that CAPE reduces acute ROS production by activating on mitochondrial uncoupling protein UCP2. To determine whether UCP2 modulated CAPE effects on IEGs expression we used both UCP2 siRNA and Genipin, a pharmacological inhibitor of UCP2, to inhibit UCP2 expression or activation. After UCP2 siRNA transfection, expression of UCP2 mRNA was significantly inhibited, and CAPE effects on balancing ARC, EGR1, FOS, JUNB, and JUND expression during oxidative stress were aborted (Figure 8A–E). A similar trend was observed when UCP2 was pharmacologically inhibited by Genipin (Figure 9A–E). Both methods suggest that the actions of CAPE on IEG expression are mediated by UCP2 (Figure 8F and Figure 9F).

### 2.9. UCP2 Deficiency Interrupts the Inhibitory Actions of CAPE on p38 MAPK/CREB Activation during Oxidative Stress

**Figure 10:**
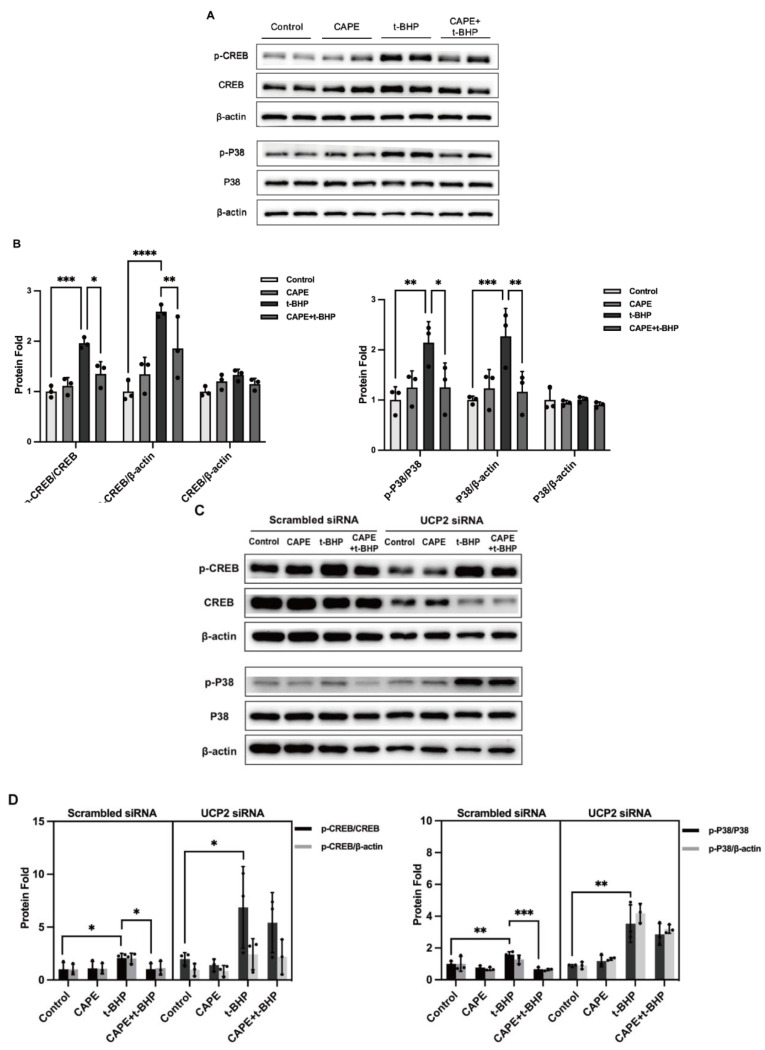

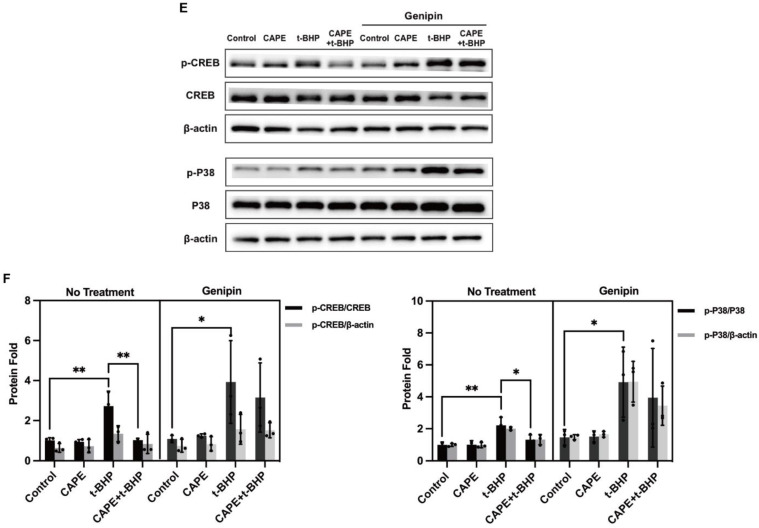
p38 MAPK/CREB signaling pathway is controlled by CAPE and during oxidative stress. p38 MAPK and CREB activation after ARPE−19 cells were treated with CAPE after exposure to t-BHP(**A**), UCP2 siRNA (**C**) Genipin (**E**). Densitometric quantification of relative protein (**B**,**D**,**F**). Experiments were repeated 3 times. β-actin was used as a loading reference. * *p* < 0.05, ** *p*< 0.01, *** *p* < 0.001, **** *p* < 0.0001.

To investigate mechanisms transmitting UCP2 signals on IEG expression we examined control by the p38MAPK/CREB pathway which is intimately associated with IEGs. The phosphorylation levels of p38 and CREB were significantly upregulated in the oxidative stress environment, and activation of this pathway was inhibited by CAPE (Figure 10A,B). Conversely, knockdown or pharmacological inhibition of UCP2 expression blocked the inhibitory effects of CAPE on p38/MAPK-CREB activation (Figure 10C–F).

## 3. Discussion

Oxidative stress is closely linked to several neurodegenerative ophthalmic diseases, including AMD, glaucoma, and diabetic retinopathy [26]. Increased oxidative stress and decreased antioxidant levels may contribute synergistically to disease development. As a tissue with high metabolic demand and ROS production, retinal cells, especially the RPE cells, are susceptible to oxidative insults [27]. The melanin pigment granules in RPE cells are the primary sites of light energy absorption, and the RPE cells are in close proximity to the highly oxygen-containing choroidal blood supply, which makes them particularly vulnerable to oxidative stress. Development of therapeutics that reduce oxidative insults to halt or delay retinal disease progression are therefore intensely pursued.

Compounds with antioxidant properties can mitigate the effects of oxidative insults [28]. CAPE, a major ingredient of propolis, and is responsible for most of its antioxidant benefits [29]. Polyphenols with hydroxyl groups in the catechol ring of CAPE provide strong antioxidant properties and affect a range of biological pathways [30,31], which has been demonstrated in many cell types against oxidative stimulus [32,33,34]. Among a series of structural mimetics, we found that CAPE had the greatest impact on the generation of mitochondrial ROS. Though unlikely, we cannot exclude the possibility that the weak activity seen with some of the compounds tested was unique to the cell type tested. CAPE was able to inhibit production of both cellular and mitochondrial ROS generation during oxidative stress induced by t-BHP (Figure 1). This study, as a confirmation and extension of our earlier results [21], suggests that CAPE has actions in addition to the general anti-oxidant properties of its polyphenol structure.

ROS production is thought to trigger a series of biochemical events that ultimately contribute to apoptosis [35]. Such cell death in the RPE may be critical in the development of ARMD. In neurons, CAPE diminishes apoptosis dysregulation to preserve the number of neurons by suppressing inflammation, mitochondrial cytochrome c release, and ROS production [31]. In an optic nerve crush rat model, attenuated inflammatory responses in Müller cells can protect retinal ganglion cells from death [19]. CAPE treatment decreases oxidative stress in retinas of the STZ-induced diabetic rat model [20], protects 661 W cells from oxidative injury-induced cell death by promoting expression of the antioxidative marker HO−1, and enhances electroretinography responses in dim-reared albino rats [36]. Similarly, in our study CAPE prevented oxidative stress-induced apoptosis in ARPE−19 cells as measured by Annexin V-FITC/PI labeling (Figure 2).

Loss of the junction protein ZO−1, and subsequent breakdown of the blood–retinal barrier, are found in a number of retinal diseases, particularly diabetic retinopathy [37]. CAPE reversed the oxidative injury-induced ZO−1 loss in a time-dependent manner and restored ARPE−19 cell morphology and tight junction structure, which suggests that CAPE is likely to preserve the barrier function of the RPE, and probably in the retinal vascular network where junction disassembly and leakage are features of retinal diseases (Figure 3). Thus, the above result points to a promising role for CAPE in retinal neuroprotection by maintaining RPE layer integrity.

To elucidate downstream molecular mechanisms of CAPE, we compared global ARPE−19 gene expression in the oxidative stress versus CAPE treatment and control groups and identified a substantial number of genes whose expression was regulated by oxidative stress and reversed by CAPE back to near control levels (Figure 4). An important cluster of genes responding in this way includes a group of IEGs (ARC, EGR1, FOS, JUNB, JUND) as validated in qPCR (Figure 5). IEGs are a group of genes expressed early in response to distinct extracellular stimuli such as growth factors, cytokines, tumor promoters, hormones, and stress. These include the AP−1 gene family (FOS and JUN), EGR, and MYC. Most of these encode transcription factors that regulate genes involved in normal cell growth and differentiation, intracellular information transmission, and energy metabolism [38]. ARC, EGR1, and FOS showed a large quantitative response (up to hundreds or thousands-fold elevation) when exposed to oxidative insults, while JUNB and JUND transcription induction showed smaller increases (up to ~10-fold elevation) over control levels then showed a declining pattern with increased exposure time. These results suggest that IEGs’ regulation is rapid and large in response to oxidative stress but refractory with increased exposure time, while the effect of CAPE is to reverse these changes. Other well-characterized IEGs like ZNF268 and MYC were increased by oxidative stress, but not regulated by CAPE.

IEGs are known to be critical for the structure, function, and plasticity of both excitatory and inhibitory synapses across various cell types. Distinct IEGs are influenced in a pathway- and stimulus-specific manner on synapse development and plasticity to regulate the function of neurons [39]. For example, hyperglycemia induces abnormal IEG expression and is involved in prothrombotic and proinflammatory pathways in the retina vasculature [40], inflammatory marker expression in microglia, and astrocytes in STZ diabetic mouse retinas [41]. Involvement of IEGs in retinal degenerative conditions may lend to development of new and unique therapies. Transcripts of IEGs are rapidly induced in response to acute stress or proliferation-inducing signals. As transcription factors, rapid IEGs’ expression responds to cellular stimulation and participates in cellular signaling pathways. The protein products of IEGs can form dimers and regulate apoptosis in both nonneuronal and neuronal cells. In this study, we report a selective and abnormally sustained induction of IEGs in cultured ARPE−19 cells under oxidative insults. In addition, we describe the lack of induction of IEGs after CAPE pretreatment that is relatively resistant to apoptosis. As has been suggested by others, a prolonged time course of IEGs’ expression in this instance would allow for the formation of heterodimers with a series of successively transcribed gene products, potentially controlling a complex set of regulatory events for multiple target genes [42].

IEG activation is highly complex and involves various promoter elements and transcription factors. Serum response-(SREs) and cAMP-response elements (CREs) are typically found in the promoters of IEGs. The cAMP-responsive element-binding protein (CREB) has been the most studied in regulating neuronal behaviors. The activation of IEGs promoters occurs via phosphorylation of mitogen-activated protein kinase (MAPK), protein kinase A (PKA), RhoA-actin, phosphor-inositide 3 kinase (PI3K), nuclear factor-kappa B (NF-kB) or other stress-induced kinases [43]. Generally, inducible IEG expression is triggered by MAPK cascades: the RAS-MAPK pathway, which promotes ERK1/2 activation by growth factors and mitogens, or the p38-MAPK pathway, which is activated by stress. Enrichment analysis of DEGs showed multiple cellular components, biological processes, and molecular functions were under the regulation of oxidative stress and CAPE in opposite directions. For instance, the abnormal MAPK tyrosine phosphorylase activity after oxidative injury was reversed by CAPE (Figure S2).

We recently reported that a key action of CAPE on ROS production was via activation of mitochondrial UCP2 [21], the main source of intrinsic ROS generation. It is possible to achieve the optimal balance between energy production and ROS production by carefully titrating UCP2 activity using a wide variety of regulatory compounds [44]. We now show that oxidative insults promote phosphorylation of p38-MAPK and CREB, while CAPE suppressed their activation and that inhibition of UCP2 expression or activity aborts CAPE’s effect on this signal pathway. Our finding agrees with previous findings that oxidative stress-induced RPE cell injury is associated with the p38-MAPK signaling pathway [45]. Proof of principle experiments in preclinical models have shown the neuroprotective effects of p38-MAPK inhibitors, and UCP2 is a negative regulator of oxidative insult via p38-MAPK signaling networks [46,47]. These results denoted the mechanism of RPE vulnerability to oxidative injury and CAPE increasing its survival.

At present we do not know whether the activity of UCP2 controlling ROS levels is sufficient to regulate p38-MAPK, CREB, and transcription of IEGs and other genes, or whether UCP2 activity is also linked to other mitochondrial pathways. Understanding these mechanisms to target IEGs expression and function remains an untapped area in retinal neurodegenerative diseases [48]. Our results further support the hypothesis that UCP2 is a key molecule regulating oxidative stress-induced injury in ARPE−19 cells, and further studies may lead to new treatments for retinal neurodegenerative diseases.

## 4. Materials and Methods

### 4.1. Antibodies and Reagents

Chemicals. CGA, TCA, IQT, and CUM were purchased from Thermo Fisher Scientific (Waltham, MA, USA) unless otherwise noted. The tert-Butyl hydroperoxide solution (t-BHP) was obtained from Sigma-Aldrich (St. Louis, MO, USA). Caffeic acid phenethyl ester (CAPE) was purchased from Santa Cruz Biotechnology (Dallas, TX, USA). UCP2 inhibitor Genipin was obtained from MedChem Express (Monmouth Junction, NJ, USA). The primary antibodies used were as follows: CREB, Phospho-CREB, p38, Phospho-p38, β-actin (Cell Signaling Technology, Danvers, MA, USA); ZO−1 (Proteintech, Rosemont, IL, USA). Information on other reagents and antibodies is shown in Supplementary Table S1.

### 4.2. Human Retinal Pigment Epithelial (ARPE−19) Cells Culture and Treatment

ARPE−19 cells were purchased from the American Type Culture Collection (Manassas, VA, USA), and cultured in T25 flasks using 10%FBS, 1% penicillin-streptomycin(*p*/S) solution in DMEM/F12 basic culture medium (Gibco, Grand Island, NY, USA) at 37 °C and 5% CO_2_.

In all experiments, ARPE−19 cells used were 4–10 passages. Cells were seeded in 6-well (2 × 10^5^ cells/well), 12-well (1 × 10^5^ cells/well), or 96-well (5 × 10^3^ cells/well) plate. After 12 h, the confluence reached 80–90%, and cells were washed with DPBS (Gibco, Grand Island, NY, USA). Cells were starved in Fetal Bovine Serum (FBS)-free DMEM/F12 (without phenol red) culture medium (Gibco, Grand Island, NY, USA) for 6 h. CAPE or t-BHP was also diluted in FBS-free DMEM/F12 (without phenol red) culture medium. ARPE−19 cells were pretreated with 200 μM Genepin for 2 h. After 2 h 20 μM CAPE treatment, ARPE−19 cells were stimulated with 200 μM t-BHP accordingly. Other reagents (CGA, TCA, IQT, and CUM) were diluted and applied in the same way as CAPE.

### 4.3. RNA-Sequencing

ARPE19 cells were treated with 20 μM CAPE for 2 h and subsequently stimulated with 200 μM t-BHP for 12 h. RNA was extracted and purified with EZ-press RNA Purification Kit (EZBioscience, Roseville, MN, USA) following the manufacturer’s instructions. After analyzing RNA sample quality, the cDNA libraries were synthesized and sequenced by MGI Tech (Shenzhen, China) with the BGISEQ platform. In brief, the quality of the RNA samples was assessed by an Agilent Bioanalyzer (Agilent, Santa Clara, CA, USA). Single-strand circular DNA libraries were prepared with MGI easy PCR free library preparation kit (MGI Tech, Shenzhen, China) to generate single-strand long fragment DNA and form a DNA nano ball (DNB) structure with rolling circle amplification (RCA) technology. Each DNB was loaded on flowcell (FCL) and sequenced using MGI-seq 2000 (MGI Tech, Shenzhen, China) with combinatorial prober anchor synthesis (cPAS) technique to obtain Fastq data.

All samples were tested using the DNBSEQ platform. This project used the filtering software SOAPnuke (v1.5.2) developed by BGI independently for filtering with the “Parameters -l 15 -q 0.2 -*n* 0.05” option. After quality control, the filtered clean reads were aligned to the reference sequence (Homo_sapiens, NCBI, Reference Genome Version: GCF_.39_GRCh38.p13). Hierarchical Indexing for Spliced Alignment of Transcripts (HISAT2, v2.0.4) software was used for mapping RNA-seq reads. Perl script was used for sequencing saturation analysis. For expression quantification, we used Bowtie2 (v2.2.5) to map the clean reads to the reference gene sequence (transcriptome), and then used RSEM (v1.2.8) to calculate the gene expression level of each sample. Each group has two replicate samples. The PossionDis method (Parameters: Fold Change >= 2 and FDR <= 0.001) was applied to screen differentially expressed genes between two samples, and the DEseq2 method (Parameters: Q value or Adjusted *p*-value <= 0.05) was applied to describe the genes detection of different samples with a different expression. According to the results of differential gene detection, the R package heatmap was used to perform hierarchical clustering analysis on the union set differential genes. The average transcripts per million (TPM) value of each group was counted for further analysis. The significant levels of terms and pathways were corrected by FDR-adjusted *p*-value (q-value), with a rigorous threshold (q ≤ 0.05) by Bonferroni as previously described [49]. All analyses were performed on the Dr. Tom analysis system constructed by MGI.

GO enrichment analysis includes three categories: molecular function, cellular component, and biological process. Functional classification is conducted based on DEGs test results. There are sub-categories for each level under each major category. GO annotation of DEGs is classified and mapped. According to the GO annotation results and the official classification, functionally classify the DEGs. At the same time, the phyper function in R software is used for enrichment analysis, calculating the *p*-value, and the Q-value was obtained by correction of the *p*-value. Generally, functions with Q-value <= 0.05 are considered significant enrichment.

### 4.4. Quantitative Real-Time PCR (qPCR)

Cells were pretreated with or without 20 μM CAPE for 2 h and stimulated with 200 μM t-BHP for 45 min, 2 h, 4 h, 8 h, 12 h, and 24 h in sequence. After RNA extraction and purification, final RNA concentrations were determined using a NanoDrop 2000 Spectrophotometer (Thermo Fisher Scientific, Waltham, MA, USA). The cDNA was synthesized with Reverse Transcription Master Mix (EZBioscience, Roseville, MN, USA) according to the manufacturer’s protocol. Primers were purchased from Sangon Biotech (Shanghai, China). The sequence information is listed in Supplementary Table S2. For quantitative real-time PCR we used EZ-press One Step qRT-PCR Kit (EZBioscience, Roseville, MN, USA). Samples in triplicate were run in 384-well plates on Light Cycler 480 II System (Roche, Basel, Switzerland). The relative expression level for each gene was calculated by the 2^−ΔΔCt^ method and normalized to Polr2f. Genes were considered upregulated or downregulated if *p* < 0.05.

### 4.5. Western Blot

After treatment, culture medium was aspirated and cells washed with 1 × PBS (Solarbio, Beijing, China). Cells were lysed by adding 100 µL RIPA lysis buffer containing 1% proteinase inhibitor (Target Mol, Shanghai, China) and 1% phosphatase inhibitor (Beyotime, Shanghai, China) per well of 6-well plate. Cells were immediately scraped off the plate and transferred to a microcentrifuge tube. Tubes were placed on ice and samples sonicated for 10–15 s to complete cell lysis. Samples were centrifuged at 12,000× *g* for 20 min, and the supernatants were collected. Protein was quantified by a BCA kit (Solarbio, Beijing, China) according to the manufacturer’s instructions. An amount of 20 ul 5×SDS-PAGE loading buffer (Solarbio, Beijing, China) was added to 80 ul sample supernatant per tube and boiled at 100 °C for 5 min, then 20 ug protein of each sample was loaded, electrophoresed, and transferred onto PVDF (Roche, Basel, Switzerland) membranes, and blocked with QickBlock^TM^ Western Block Buffer (Beyotime, Shanghai, China) at room temperature (RT) for 15 min. The primary antibodies p38 (1:1000), *p*-p38 (1:1000), CREB (1:1000), *p*-CREB (1:1000), or β-actin (1:2000) were incubated overnight at 4 °C. The membranes were washed in 1 × TBST buffer. After which, the membranes were incubated with horseradish peroxidase (HRP)-linked second antibody (1:2000) for 2 h at RT. Washing membranes after incubation and adding enhanced chemiluminescence (ECL) reagents to detect immunoblots. The reactions were visualized on Tanon 4800 (Tanon, Shanghai, China) and semi-quantified by densitometry measurement obtained using ImageJ (Version 2.1.0, National Institutes of Health, Bethesda, MD, USA) with normalization to internal control β-actin.

### 4.6. Transient Transfection

ARPE19 cells were transfected with UCP2 siRNA or Scrambled siRNA (Santa Cruz Biotechnology, Dallas, TX, USA) at 70–80% confluence by using the jetPRIME (Polyplus Transfection, Illkirch, France) in accordance with the manufacturer’s protocol. Briefly, 200 μL jetPRIME buffer containing 50 nM siRNA and 10 μL jetPRIME reagent was added to one well in a 6-well plate and transfected for 36 h. The silencing efficiency was evidenced by qPCR. The detailed siRNA information is provided in Supplementary Table S3.

### 4.7. Immunolabeling

Cells were seeded in a 35 mm glass bottom dish (Cellvis, Mountain View, CA, USA) at 80–90% confluence. After treatment, cells were washed with 37 °C pre-warmed HBSS. Cells were fixed with 4% PFA for 15 min at 37 °C, followed by rinsing 3 times with 1×PBS for 5 min each. Cells were permeabilized with 0.2% TritonX−100 in 1 × PBS for 10 min at 37 °C, followed by rinsing 3 times with 1 × PBS for 3 min each. Cells were incubated with 5% goat serum in 1×PBS blocking buffer at room temperature for 1 h. Primary antibody ZO−1(1:2000) was diluted in 1%BSA in 1 × PBS antibody dilution buffer and incubated at 4 °C overnight. After washing 3 times with 1 × PBS for 5 min each, samples were incubated with fluorochrome-labeled goat anti-rabbit secondary antibody (1:500) (Alexa Fluor 488, Abcam, Boston, MA, USA) at RT for 2 h. After washing 3 times with 1 × PBS for 5 min each, samples were mounted with Fluoroshield™ with DAPI (Sigma-Aldrich, St. Louis, MO, USA). Images were taken and processed using a confocal microscope (Zeiss, Baden-Württemberg, Germany).

### 4.8. Reactive Oxygen Species (ROS) Determination

To measure ROS level in cells, we detected mitochondrial ROS by applying MitoSOX™ Red mitochondrial superoxide indicator (Invitrogen, Carlsbad, CA, USA) at ~510/580 nm, and detected cellular oxidative stress with CellROX™ Deep Red Reagent (Invitrogen, Carlsbad, CA, USA) at ~644/665 nm in live cells according to the manuals. Briefly, after treatment we loaded 5µM MitoSOX™ working solution to cover cells and incubated for 10 min at 37 °C, protected from light. Similarly, for CellROX™ we loaded a 5µM working solution and incubated cells for 30 min at 37 °C, protected from light. Counterstained nuclei with Hochest 33,342 (Beyotime, Shanghai, China) for 10 min at 37 °C, protected from light. We mounted cells in warm buffer for live cell imaging with confocal microscope (Zeiss, Baden-Württemberg, Germany).

### 4.9. Analysis of Apoptosis

Apoptotic ARPE−19 cells were identified by staining with Annexin V-fluorescein isothiocyanate (FITC) and Propidium iodide (PI) using Annexin V-FITC/PI Apoptosis Detection Kit (Beyotime, Shanghai, China). After stimulation, cells were treated with 100 µL of binding buffer containing 1 µL of Annexin V-FITC and 10 µg/mL PI for 15 min in the dark, after which 400 µL of binding buffer was added and subjected to flow cytometry; Or, after staining directly observe the fluorescence under microscope (Zeiss, Baden-Württemberg, Germany) according to the manufacturer’s manual. Flow cytometry analysis was performed using the FACSCanto II cytometer (BD FACSCanto II, San Diego, CA, USA). Analysis of data was performed using the FACSDiva software (BD, San Diego, CA, USA).

### 4.10. Statistical Analysis

Statistical comparisons were all conducted in Prism 9 software (Version 9.3.1, GraphPad Software, La Jolla, CA, USA). All data in the study were expressed as mean ± standard deviation (SD). The Shapiro–Wilk test was performed to verify the normal distribution of variables. Data were evaluated for statistical differences amongst groups using student’s 2 tailed-t-test and a two-way analysis of variance (ANOVA) followed by Bonferroni’s post hoc comparison. The *p* values < 0.05 were considered statistically significant.

## 5. Conclusions

In conclusion, our results reveal a potent protective role of CAPE against oxidative stress-induced apoptosis in ARPE−19 cells and show that this effect was mediated by UCP2 via regulating CREB/p38-MAPK/IEGs signal. Since systemic application of CAPE has been shown to prevent ischemia-reperfusion damage to retinal ganglion cells in mice, this compound is likely to be a potent protective agent for a variety of retinal degenerative diseases and may have strong clinical potential as a therapeutic agent.

## Data Availability

The data presented in this study are available on request from the corresponding author.

## Supplementary Materials

The following supporting information can be downloaded at: https://www.mdpi.com/article/10.3390/ijms24043565/s1.
