# Supplementary material for: Molecular Mechanisms of Oxidative Stress Relief by CAPE in ARPE−19 Cells
Source: Int J Mol Sci. 2023 Feb 10;24(4):3565. doi: 10.3390/ijms24043565 (PMC9959600; doi:10.3390/ijms24043565)
Supplement: Supplementary file 1 [file ijms-24-03565-s001.zip › ijms-2133487-supplementary.pdf]

Table S1. Main materials and reagents.

Reagents	Source
Fetal Bovine Serum (FBS)	Gibco (Gibco, Grand Island, NY, USA)
Trypsin-EDTA (0.25%)	Gibco (Gibco, Grand Island, NY, USA)
DMEM/F−12 Basic	Gibco (Gibco, Grand Island, NY, USA)
DMEM/F−12 (HEPES$^-$, Phenol Red$^-$)	Gibco (Gibco, Grand Island, NY, USA)
DPBS(Ca^{2+} or Mg^{2+} free)	Gibco (Gibco, Grand Island, NY, USA)
20×TBST	Solarbio (Beijing, China)
Restore™ PLUS Western Blot Stripping Buffer	Thermo Scientific (Waltham, MA, USA)
HRP-labeled Goat Anti-Mouse IgG(H+L)	CST (Danvers, MA, USA)
HRP-labeled Goat Anti-Rabbit IgG(H+L)	CST (Danvers, MA, USA)
ECL Kit	Cyvita (Shanghai, China)
384 Well Plate	Roche (Basel, Switzerland)
4% Paraformaldehyde (PFA)	Solarbio (Beijing, China)
BSA (Fatty Acid and IgG Free, BioPremium)	Beyotime (Shanghai, China)
Goat Serum	Beyotime (Shanghai, China)
Hanks' Balanced Salt Solution (HBSS)(with Ca^{2+} and $Mg2^{+}$)	Beyotime (Shanghai, China)

Table S2. Primers for Quantitative Real-Time PCR.

Primer	Sequence
**Human Polr2f Forward.**	ACAGATCCTCTGCTCATTGC
Human Polr2f Reverse	GTGCCGCTGAGTAAATGGGA
Human ARC Forward	CTCAGCTGCAGGAAGGACAT
Human ARC Reverse	CAGTGGACAAGGCAGAACGA
Human EGR−1 Forward	GGTCAGTGGCCTAGTGAGC
Human EGR−1 Reverse	GTGCCGCTGAGTAAATGGGA
Human FOS Forward	CACTCCAAGCGGAGACAGAC
Human FOS Reverse	AGGTCATCAGGGATCTTGCAG
Human JUNB Forward	ACGACTCATACACAGCTACGG
Human JUNB Reverse	GCTCGGTTTCAGGAGTTTGTAGT
Human JUND Forward	TCATCATCGAGTCCAACGG
Human JUND Reverse	TTCTGCTTGTGTAAATCCTCCAG
Human MYC Forward	GTCAAGAGGCGAACACACAAC
Human MYC Reverse	TTGGACGGACAGGATGTATGC
Human ZNF268 Forward	AGGGTCCGGACAGCTTCTAT
Human ZNF268 Reverse	GAGGTTGCAGACCAGGAGTC
Human UCP2 Forward	GTCCGGTTACAGATCCAAGGAG
Human UCP2 Reverse	CTTTACCACATCCGTGGGCT

Table S3. The sequences of the UCP2 siRNA (Human).

Product number	Sense	Antisense
sc−A	GAAGAACGGGACACCUUUATT	UAAAGGUGUCCCGUUCUUCTT
sc−B	CUCGUCUUGUUGCUGAUUATT	UAAUCAGCAACAAGACGAGTT
sc−C	GCAUCAUGGUUGGGUUCAATT	UUGAACCCAACCAUGAUGCTT

A

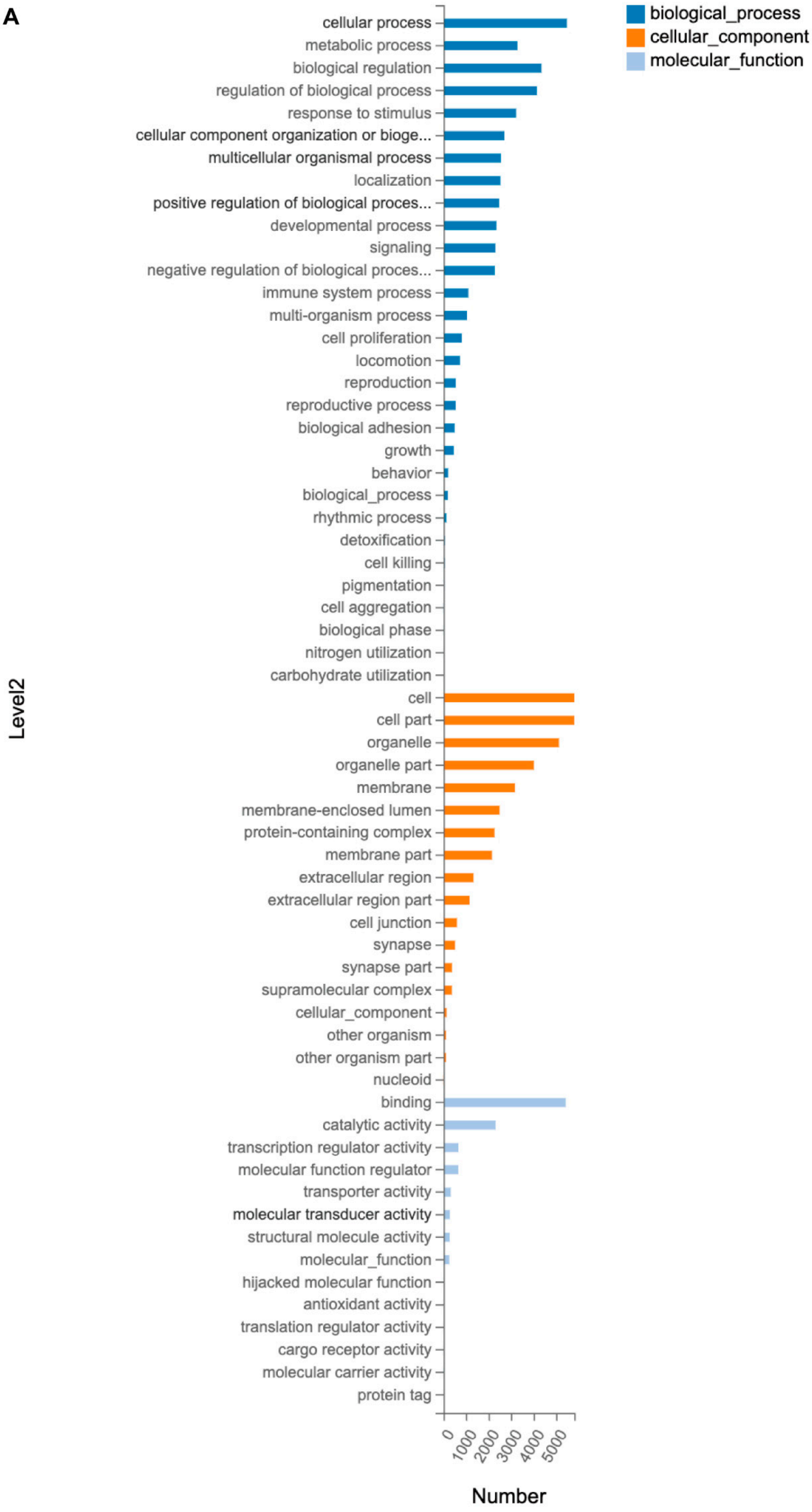

t-BHP vs Control

Figure S1. GO enrichment of DEGs. Biological process, cellular component, and molecular function classification were conducted based on DEGs test results of the t-BHP vs. control group (**A**), or DEGs of the CAPE + t-BHP vs. control group (**A**). GO annotation of DEGs is classified and mapped. *X*-axis: the number of genes annotated to the GO entry; *Y*-axis: the GO functional classification.

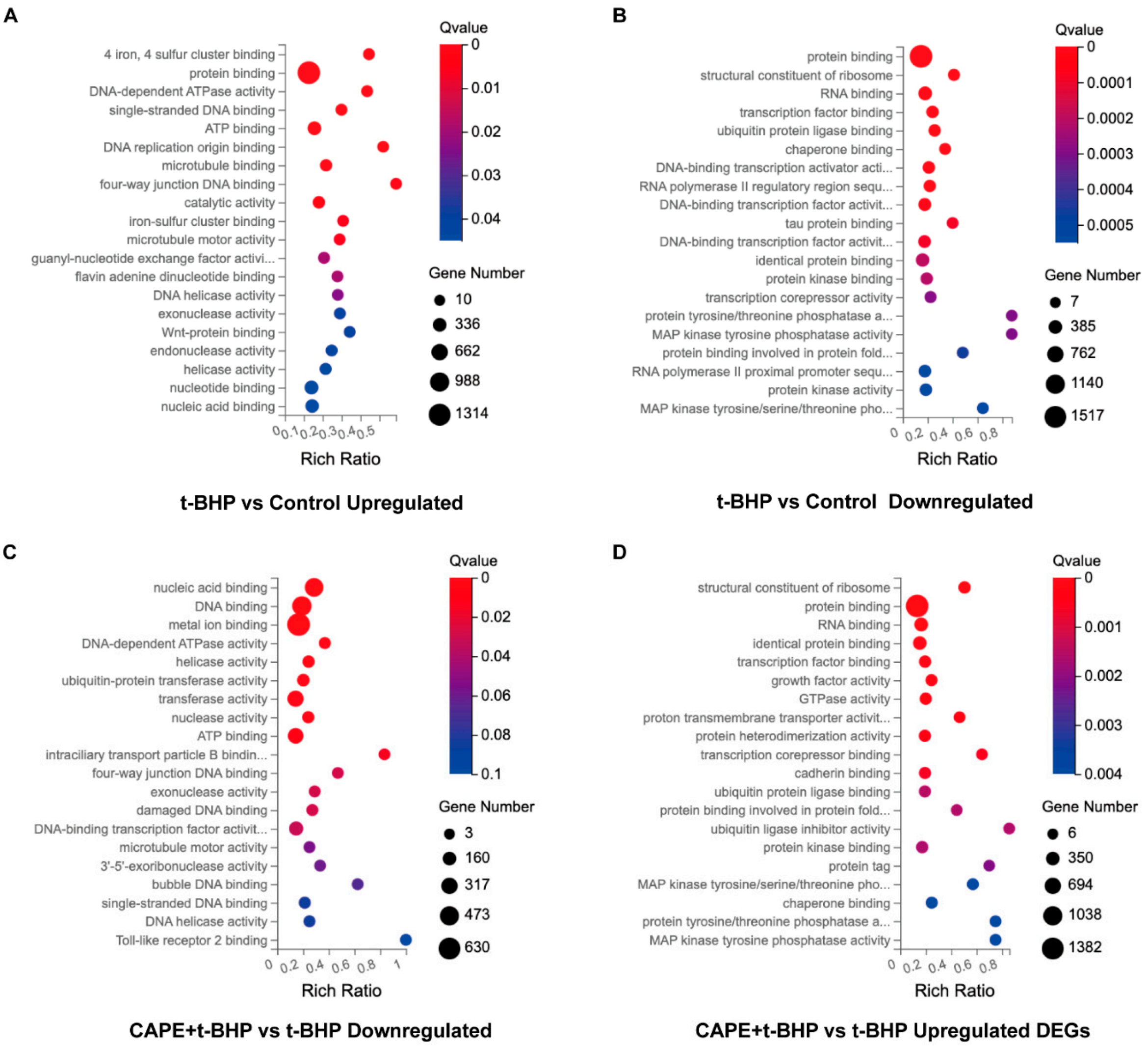

Figure S2. The top 20 of GO enrichment of DEGs in molecular function. Upregulated (**A**) or downregulated (**B**) DEGs of t-BHP vs. control enrichment; Downregulated (**C**) or upregulated (**D**) DEGs of CAPE + t-BHP vs. t-BHP enrichment. Q-value <= 0.05 are considered significant enrichment.

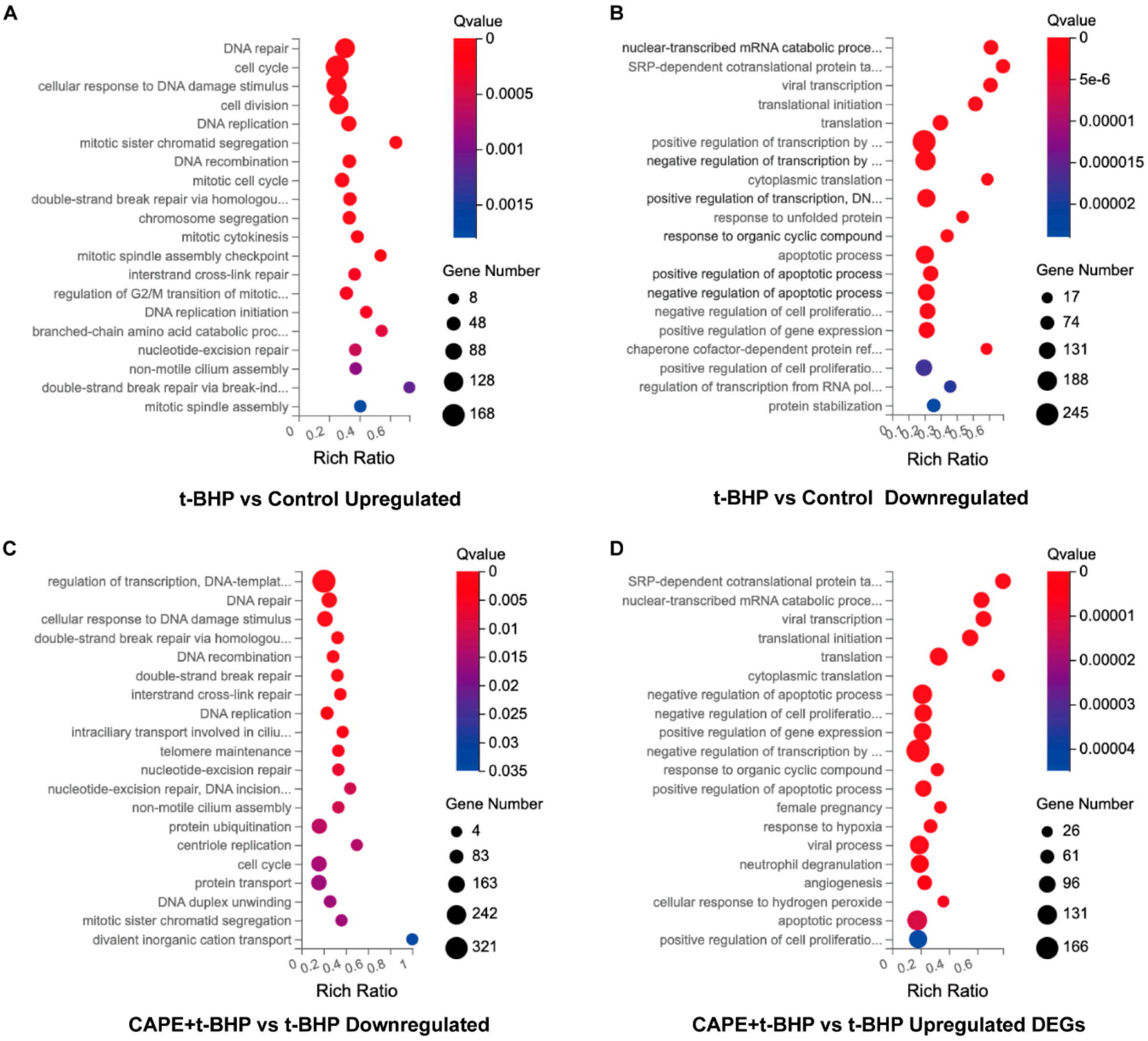

Figure S3. The top 20 of GO enrichment of DEGs in biological process. Upregulated (**A**) or downregulated (**B**) DEGs of t-BHP vs. control enrichment; Downregulated (**C**) or upregulated (**D**) DEGs of CAPE + t-BHP vs. t-BHP enrichment. Q-value <= 0.05 are considered significant enrichment.
